# Maize yield response to a phosphorus-solubilizing microbial inoculant in field trials

**DOI:** 10.1017/S0021859614001166

**Published:** 2014-11-14

**Authors:** M. LEGGETT, N. K. NEWLANDS, D. GREENSHIELDS, L. WEST, S. INMAN, M. E. KOIVUNEN

**Affiliations:** 1 Novozymes BioAg Ltd., 3935 Thatcher Avenue, Saskatoon, SK S7R 1A3, Canada; 2 Science and Technology Branch (S&T), Agriculture and Agri-Food Canada (AAFC), Lethbridge Research Centre, 5403 1st. Ave. S., P.O. Box 3000, Lethbridge, AB T1J 4B1, Canada; 3 Novozymes Biologicals Inc., 5400 Corporate Circle, Salem, VA 24153, USA; 4 Gowan Company, LLC, 370 S. Main Street, Yuma, AZ 85364, USA; 5 College of Agriculture, Plumas Hall 104 California State University, Chico, CA 95929-0310, USA

## Abstract

Findings from multi-year, multi-site field trial experiments measuring maize yield response to inoculation with the phosphorus-solubilizing fungus, *Penicillium bilaiae* Chalabuda are presented. The main objective was to evaluate representative data on crop response to the inoculant across a broad set of different soil, agronomic management and climate conditions. A statistical analysis of crop yield response and its variability was conducted to guide further implementation of a stratified trial and sampling plan. Field trials, analysed in the present study, were conducted across the major maize producing agricultural cropland of the United States (2005–11) comprising 92 small (with sampling replication) and 369 large (without replication) trials. The multi-plot design enabled both a determination of how sampling area affects the estimation of maize yield and yield variance and an estimation of the ability of inoculation with *P. bilaiae* to increase maize yield. Inoculation increased maize yield in 66 of the 92 small and 295 of the 369 large field trials (within the small plots, yield increased significantly at the 95% confidence level, by 0·17 ± 0·044 t/ha or 1·8%, while in the larger plots, yield increases were higher and less variable (i.e., 0·33 ± 0·026 t/ha or 3·5%). There was considerable inter-annual variability in maize yield response attributed to inoculation compared to the un-inoculated control, with yield increases varying from 0·7 ± 0·75 up to 3·7 ± 0·73%. No significant correlation between yield response and soil acidity (i.e., pH) was detected, and it appears that pH reduction (through organic acid or proton efflux) was unlikely to be the primary pathway for better phosphorus availability measured as increased yield. Seed treatment and granular or dribble band formulations of the inoculant were found to be equally effective. Inoculation was most effective at increasing maize yield in fields that had low or very low soil phosphorus status for both small and large plots. At higher levels of soil phosphorus, yield in the large plots increased more with inoculation than in the small plots, which could be explained by phosphorus fertilization histories for the different field locations, as well as transient (e.g., rainfall) and topographic effects.

## INTRODUCTION

Agricultural systems rely on fertilizer inputs to supplement soil nutrient levels to help maximize crop yield (Shen *et al.* 2011). While in many agricultural areas, the lack of available nutrients limits crop yields, in others fertilizers are being over-used and misapplied (i.e. non-optimal crop uptake due to misplacement in location and/or timing). Overuse of nitrogen (N) and phosphate (P_2_O_5_) fertilizers can cause significant environmental degradation and pollution through a wide array of inter-related physical, chemical and biological pathways (e.g., soil acidification, leaching into surface and ground water). Worldwide interest in the use of biological agents, such as phosphorus (P)-solubilizing microorganisms is increasing because they offer significant improvements for crop growth and development, while providing new opportunities for simultaneous bio-control and reduction in detrimental environmental impacts linked with overuse of phosphate fertilizers (Vassilev *et al.* 2006). Gerretsen (1948) demonstrated a direct contribution by P-solubilizing bacteria and fungi to improved growth of oats, rye, mustard and rape. More recent studies report increased crop yields in response to inoculation with soil microorganisms associated with improved plant/crop phosphate nutrition – with hundreds of P-solubilizing fungi and bacteria already isolated and identified (Rodriquez & Fraga 1999; Khan *et al.* 2007; Harvey *et al.* 2009). While there is significant evidence for the increased dissolution of inorganic phosphorus by microbial communities *in vitro*, the performance of phosphorus-solubilizing microbes *in situ* has been contradictory (Khan *et al.* 2010). Also, very few inoculants have been tested in agricultural field trials to determine their effectiveness in increasing crop yield. Hence, more field trials on P-solubilizing inoculants are needed under a wide variety of soil and environmental conditions.

Phosphorus is the most important plant macronutrient second to nitrogen, and it plays a role in many aspects of plant structure and function. Phosphorus is chemically reactive and readily converted to other, less soluble forms depending on soil acidity, temperature, moisture and other factors. The majority of P in soil is immobile and sparingly soluble, making it unavailable to plants. For this reason, P is applied at regular intervals, depending on soil characteristics and crop rotations, to try to maintain optimum levels for crop production, thereby increasing the risk of its build-up in agricultural soils (Stevenson & Cole 1999). The majority of fertilizer P is mined from non-renewable rock phosphate and conservation of P is quickly becoming a necessity because worldwide stocks are generally of insoluble forms, immobile and unavailable to crops (Cordell *et al.* 2009), and low P availability can be the limiting factor causing crop yield loss (Zhu *et al.* 2005; Calderón-Vázquez *et al.* 2011; Lynch 2011). It is critical to optimize P applications, as runoff of fertilizer P bound in bulk soil is a major contributor to freshwater eutrophication (Carpenter *et al.* 1998). One can increase the uptake of P and yield of maize by increasing P concentration and/or the activity of root-zone soil microbes. There are many pathways leading to higher availability of P in the rhizospheres, but primary pathways involve root growth promotion and/or rhizosphere acidification through hydrolytic enzymes, organic acids and siderophores (iron-metabolizing compounds secreted by microorganisms) (Richardson & Simpson 2011).

Inoculant use in maize (*Zea mays* L.) is of particular interest to both scientists and the agri-industry, because maize is among the most widely cultivated crops worldwide. It is also seeded on more acres than any other crop in the United States (USA). Maize has a particularly high nutrient requirement to meet genetic potential for growth and yield. Furthermore, even though annual maize yield incremental increases are small, such increases still have the potential to provide substantial economic benefits for farmers and environmental gains for society. Inoculants are relatively inexpensive to produce, and maize yield increases as small as 0·6 t/ha can provide an economic return of US$25–50/ha to farmers (Al-Kaisi & Yin 2004). Crop yield is typically variable at the field scale, which means that it must be calibrated to regional differences in climate and environmental conditions. Multi-site and multi-year field trial data are needed to obtain adequate statistical significance for separation of treatment effects and ensure robustness of analysis. Statistical robustness ensures that yield increases, no matter how large or small, are reproducible across a wide range of environmental conditions. Also, the power of an experiment depends on the size of the difference to be measured, the variability of the data and the number of replicates of each treatment. Hence, as many as 9–28 replicates may be required to detect a 10% difference between treatments (Edmeades 2002). Obtaining robust crop yield statistics remains a challenge due to both logistical constraints and the associated cost of sampling, as well as the underlying complexity of environmental factors that mediate attainable yield (i.e., in relation to theoretical/potential yield).

*Penicillium bilaiae*, a soil fungus isolated near Lethbridge, Alberta, Canada is a phosphorus-solubilizing inoculant, and solubilizes soil phosphorus by secreting citric and oxalic acids (Cunningham & Kuiack 1992). After inoculation as a seed treatment, the fungus colonizes roots. It has been shown to increase root length and root hair abundance (Gulden & Vessey 2000; Vessey & Heisinger 2001), which improves access to nutrients and moisture that crops need to maintain optimal growth. However, previous field studies conducted on various crops, in different climatic regions, cropping systems and agronomic management conditions provide conflicting results regarding the effect of *P. bilaiae* inoculation to increase crop yield (Kucey^1987^, ^1988^; Kucey & Leggett 1989; Beckie *et al.* 1998; Grant *et al.* 2000, 2002; Leggett *et al.* 2007; Karamanos *et al.* 2010). The current paper addresses the need for *in situ* testing of crop yield response to P-solubilizing inoculants across a wide variety of soil and environmental conditions. Findings from the statistical analysis of field trial data collected over multiple years across the USA to test the effect of *P. bilaiae* inoculation on maize yield are reported.

## MATERIALS AND METHODS

### Field site characteristics

**Fig. 1.**
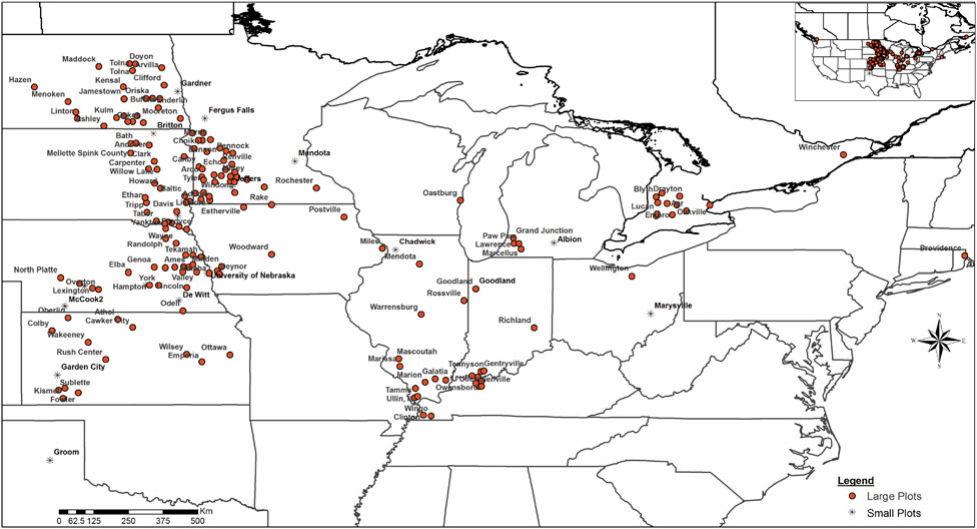
Field trial site distribution across major maize production regions within the United States (2005–11). Trials consisted of small (with replication) and large area plots (without replication). Replicated trial designs consisted of a split-plot or randomized block design with six replications per treatment. Trial locations were from 47°49′N to 35°12′N and from 38°14′W to 75° 21′W and elevations ranged from 3 to 992 m.

A total of 92 small (with sampling replication), and 369 large plot field trials (without replication) were conducted over 6 years (2005–11) at the locations shown in Fig. 1. Soil test data were obtained at 18 of the small split-plot and 116 of the large plot trial sites. Commercial and test formulations of the *P. bilaiae* inoculant called JumpStart™ (hereafter, JS) developed by Novozymes BioAg Ltd., Canada were used. Several trial types were conducted during this period (Table 1) using different JS inoculant treatments (i.e., commercial and test formulations). Table 2 provides a summary of the small and large plot trials conducted in 2005–11. Both replicated (small plots) and non-replicated (large plots) trial designs were used. Replicated studies used either a split-plot or randomized block design with six replications per treatment.

**Table 1.** Summary of different inoculation treatments applied in the small plot field trials to evaluate their relative effect on maize yield

Trial type	Design	Inoculant	Treatment	P_2_O_5_ kg/ha (seed-placed)	Field sites	Year(s)
Efficacy	Split plot	Jumpstart commercial rate*		0, 10, 20	Fergus Falls, MN; DeWitt, NE; and Jeffers MN	2006
Formulation	Split plot	Jumpstart commercial rate*	New JumpStart formulation commercial rate†	0, 10, 20, 30	Aurora, NE (2009, 2010); Centerville, SD (2009, 2010); Fergus Falls, MN (2009); Garden City KS (2009); Groom, TX (2009); Britten, SD (2010); University of Nebraska, NE (2010)	2009, 2010
Application dribble band	RCB‡	Jumpstart commercial rate*		10	DeWitt NE (2006); Fergus Falls, MN (2006, 2007, 2008); Centerville, SD (2007, 2008); Jeffers, MN (2006); Gardener, ND (2008); York, NE (2007)	2006, 2007, 2008
Application granule	RCB‡	Jumpstart commercial rate*	Granules at 1, 2 and 3 times commercial seed rate	10	DeWitt NE (2006); Fergus Falls, MN (2006, 2007, 2008); Centerville, SD (2007, 2008); Jeffers, MN (2006); Gardener, ND (2008); York, NE (2007)	2006, 2007, 2008
Miscellaneous inoculant combinations§	RCB‡	Jumpstart commercial rate*	JumpStart with and without other commercial inoculants	10	60	2006–2010

* 5 × 10^4^ colony forming units (cfu) *P. bilaiae*/seed.

† Test formulation.

‡ Randomized complete block.

§ Individual trial data not included.

**Table 2.** Summary of small and large plot trials used to measure maize yield response to inoculation, 2005–11

Type of trial	Sample size, *n*	Yield increase (t/ha ± s.e.)	95% CI*	Increase %	Paired *t*-test	*Z*-test (C† *v*. I‡)
Small plot	92	0·17 ± 0·044	0·08–0·26	1·8	Prob > t < 0·001	<0·001
Large plot	369	0·33 ± 0·026	0·28–0·38	3·5	Prob > t < 0·001	0·000

* Confidence interval.

† Control.

‡ Inoculated.

### Replicated small plot trials

Replicated small plot studies were managed by research co-operators in eight States; Illinois (IL), Indiana (IN), Kansas (KS), Minnesota (MN), Nebraska (NE), North Dakota (ND), South Dakota (SD) and Texas (TX). Ninety two small plot trials were conducted between 2005 and 2010 using either a split-plot or a randomized block design with six replications per treatment. Split-plot experiments consisted of two levels of experimental units; main plots and sub-plots called split-plots or split units. Each level of experimental units involved randomization. The assignment of block-level treatments to main plots and treatments to the split-plot units within each block or main plot was conducted randomly (Jones & Nachtsheim 2009). As in many field experiments, treatment factors were differentiated with respect to the ease with which they could be changed across experimental runs, given that treatments can be expensive and time-consuming to change, especially if an experiment is replicated many times. Therefore, different phosphate (P_2_O_5_) application levels were assigned to the blocks or main plots, and different inoculant treatments to the split-plots. Small plot size varied from 30 to 40 m^2^. The plots were seeded in May or June. In most trials, the inoculant was applied to seed in the form of spore slurry at the commercial application rate. In the dribble band treatment, inoculant was added to water to form slurry and applied in-furrow, and in the granular treatment, a JS granule was prepared by applying the inoculant in slurry form to peat granules. Granules were applied in furrow at rates of 3·3, 6·6 and 9·9 kg/ha.

Field trials in 2006 at DeWitt NE, Jeffers MN, and Fergus Falls MN in small plot sites were fertilized with 0, 10 or 20 kg/ha P_2_O_5_ applied as either mono-ammonium phosphate (11-50-0) or triple super phosphate (0-46-0). The trials tested the effects of P_2_O_5_ fertilization alone and in combination with *P. bilaiae* inoculation. Also, in 2009 and 2010, a series of formulation trials were conducted to test whether a new formulation of the inoculant could further increase yield. Field trial data were organized by year and phosphate fertilizer level. In 2009 and 2010, a 30 kg/ha phosphate treatment was included in the trial design, in addition to 0, 10 and 20 kg/ha phosphate treatments. Two sets of experiments were conducted to determine the relative effectiveness of different application/treatment methods (i.e., dribble band, granular and the standard seed treatment). Dribble band was compared to seed treatment in ten trials conducted between 2006 and 2008. The maize yield for each treatment was measured at harvest.

### Non-replicated large plot trials

Non-replicated trials (*n* = 369) were conducted on large plots within farmers’ fields. Plot size varied from 10 to 20 acres or 40 000–80 000 m^2^. The trials consisted of two treatments: a control using the farmer's standard practice and that same practice using seed treated with *P. bilaiae* (commercial product at the recommended rate).

### Statistical model and analysis

A generalized regression model equation (in matrix form) for a split-plot experiment is an extension of the standard multiple regression model, but includes a term to account for the whole-plot error, and is given by:1
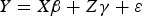
where *Y* is the *n* × *1* vector of responses (i.e., crop yield) for *n* total number of experimental runs. The matrix *X* is the *n* × *p* model matrix of factors (i.e., treatment) settings, whose *i*th row is (in a general case) the 1 × *p* polynomial model expansion, **f**(**w**_*i*_,**s**_*i*_), where ***w***_*j*_ = (*w*_*i*1_*,w*_*i*2_,…,*w*_*i,mw*_) and **s**_*i*_ = (*s*_*i*1_*,s*_*i*2_,…,*s*_*i,ms*_) denote, respectively, the settings of the *m*_*w*_ whole-plot treatments and the settings of the total *m*_*s*_ split-plot treatments for the *i*th experimental run. *β* is the *p* × 1 parameter vector of treatment effects.

For a total of *c* main plots (*c* = 92) *(k =* 1,…*c*), with *a* levels of main-plot phosphorus treatment (*a* = 4)(*i =* 1,…,*a*), and *b* levels of inoculant treatment (*b* = 3) (*j =* 1,…,*b*), the total number of experimental runs is *n* = *abc*. The second term in the above equation, combines the *n* × *b* matrix, *Z*, whose *i*th row is equal to **z**_**i,**_ an indicator variable whose value is one when the *i*th run is assigned to the *k*th main plot and zero if not. The vector, ***γ*** is a *b* × 1 vector of *main-plot* random effects, and ***ε*** *=* *(ε*_1,_ *ε*_2,_…, *ε*_*n*_)' is the *split-plot* random effects error. It is assumed that both of these errors are normally distributed whereby, ***ε~***N(0_*n*,_*σ*^2^**I**_*n*_) and ***γ~***N(0_*n*,_*σ*_*w*_^2^**I**_*c*_), where **I**_*n*_ denotes the *n* × *n* identity matrix. These errors are also assumed to be independent such that Cov(***ε, γ*) = 0**_c_ _*×*_ *_n_*. The variance-covariance matrix of the response (i.e., crop yield) vector is then:2
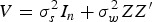


The main-plot error variance, *σ*_s_^2^, and split-plot error variance, *σ*_w_^2^, and fixed effects parameter vector, *β*, are of primary interest for statistical inference. When the split-plot experiment is balanced and the ANOVA sums of squares are orthogonal, a standard, mixed-model, ANOVA-based approach to the analysis is possible with all experimental factors or treatment settings assumed to be fixed. Thus, the split-plot trial sampling with phosphorus and inoculant treatments comprise a balanced, two-factor split-plot sampling (i.e., factorial and fractional factorial ANOVA designs), and the above generalized regression equation (Eqn 1) reduces to:3
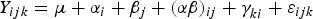
where *μ* is a constant, *α*_*i*_ denotes the *a* whole-plot treatment effects (constants) satisfying Σ_*i*_*α_i_* = 0. *β*_*j*_ denotes the *b* split-plot treatment effects (constants) satisfying Σ_*j*_*β_j_* = 0. (*αβ*)_*ij*_ denotes the *ab* interaction effects (constants) whereby Σ_*i*_(*αβ*)_*ij*_ *=* 0, for all *j,* and Σ_*j*_(*αβ*)_*ij*_ *=* 0, for all *i. γ*_*ki*_ are the *ac* whole-plot errors, and *ε*_*ijk*_ the split-plot errors.

The trial data were analysed to be consistent with this two-factor model (Eqn 3) using the ANOVA program of the JMP^®^ (version 8.0.1, 2009 SAS Institute Inc.) software package. Diagnostic verification testing was performed first, whereby all trials were checked for normality and equal variance to adhere to the modelling assumptions. In trials where the same design was used in more than 1 year and at more than one site, a combined site analysis was conducted. If the treatments were significant, the means were compared using Student's *t*-test statistics at the 95% confidence level. Sites were determined to be responsive to P_2_O_5_ application if there was a significant (*P* < 0·05) positive regression of the yield and applied P_2_O_5_. The yields of the control and inoculated treatments were measured within each site, for both the small and large plots. The percentage of trials showing increased yield due to inoculation, the average yield increase (t/ha) and the 95% confidence interval of this increase, and the percent increase was determined for both the small plot and non-replicated trials. The small plot data were partitioned by sampling year, and the large plot data were partitioned by year and location (i.e., US state). Paired observation *t*-test analysis was applied to compare mean yield increases under inoculation treatment and control across the sampling years. Relative increases in yield by location were compared where sufficient data was available (i.e., greater than 40 sites/state). The yields of the control and inoculant treatments were compared using the matched pairs program of the JMP software package. Significant differences were determined at the *P* < 0·05 level. The non-replicated trial data were segregated by state and by previous crop. Analysis of variance with contrasts was performed on field trial data to compare the increase in yield associated with JS treatments relative to an un-inoculated control. Yield and moisture values were normalized (i.e., adjustment of values measured on different scales to a nationally common scale) to enable comparisons between treatment differences.

Because phosphorus availability is linked to soil acidity, a regression analysis was conducted to determine if there was a significant (linear) correlation of yield increase and soil pH. The relative effect of inoculation on maize yield was compared, estimates across these main effects (i.e., soil type, soil phosphorus), year and location by computing the Cohen's d meta-analysis statistic. This statistic provides a common metric in standard deviation units, as the data for the set of main effects had varying sampling size (Ojiambo & Scherm 2006). Reference to these computed values of this standardized variance-based statistic, enables trials with smaller variation to be given more weight than those with larger variation. Confidence interval estimates (95% CI) of the Cohen's d effect-size statistic quantify the relative sampling effort, within each of the existing sampling sites, that would be required to increase the statistical power (relative to the 95% significance level) to ensure statistical robustness of maize yield response across differences in climate and environmental factors across the trial locations.

## RESULTS

### Yield variance across inoculant application methods

There were no differences between *P. bilaiae* inoculations on maize yield in the dribble band, granular or standard seed application, based on a sub-set of field data (i.e., 10 trials across two sampling years, 2006–08). On average, inoculation treatments increased yield by up to 0·25 t/ha (*P* < 0·05) compared to un-inoculated control treatments.

### Maize yield increase and yield variance due to inoculation

Inoculation with *P. bilaiae* increased maize yield in 66 of 92 (72%) and 295 of 369 (80%) of the small and large plots, respectively. Within the small plot trials, yield increased significantly at the 95% confidence level, by 0·17 ± 0·044 t/ha or 1·8%, while in the trials with larger plots, yield increases were almost twice as high and with less variability (i.e., 0·33 ± 0·026 t/ha or 3·5%) (Table 2). The smaller yield increases in the small plots were associated with significant inter-annual yield variability (2005–10) (Table 3). In two of the 6 years, there was a large significant effect (*P* < 0·05) in the treated (inoculated) *v.* control (un-inoculated) comparisons, with 17 of 23 (74%) trials in 2007, and 10 of 11 (91%) 2009 resulting in yield increases. The 2 years that showed a positive inoculation effect had the highest (2009, 13·7 ± 0·52 t/ha) and lowest (2007, 8·4 ± 0·84 t/ha) yields. In the larger plots, the high yield increases due to inoculation were associated with statistically significant increases in yield across all trial years (*P* < 0·01) (Table 4). Also, for both the small and large plots, the years 2007 and 2009 were associated with the lowest and highest average yields, respectively.

**Table 3.** Inter-annual variability in maize yield (2005–11) under inoculation with *P. bilaiae*, in the small plot trials, 2005–10. Yield increase (%) is for inoculated crop relative to control (not inoculated)

Year	*N*	Yield (t/ha ± s.e.)	Range	Yield increase (% ±s.e.)	95% CI*	I† *v*. C‡ (t/ha ± s.e.)	Paired *t*-test	*Z*-test (C‡ *v*. I†)
2005	10	10·1 ± 0·77	6·4–13·3	0·72 ± 0·729	−0·9 to 2·4	0·05 ± 0·081	<0·28	0·190
2006	9	11·2 ± 0·84	7·8–14·3	1·81 ± 1·131	−0·8 to 4·4	0·15 ± 0·117	<0·11	0·094
2007	23	8·4 ± 0·26	6·9–10·8	3·26 ± 1·047	1·1 to 5·4	0·28 ± 0·085	<0·00	0·001
2008	24	10·6 ± 0·44	7·3–16·2	0·62 ± 0·838	−1·1 to 2·4	0·05 ± 0·084	<0·28	0·280
2009	11	13·7 ± 0·52	10·4–15·6	3·67 ± 0·731	2·0 to 5·3	0·48 ± 0·080	<0·00	<0·001
2010	15	12·6 ± 0·57	8·5–15·4	0·68 ± 0·745	−0·9 to 2·7	0·08 ± 0·103	<0·22	0·212

* Confidence interval.

† Control.

‡ Inoculated.

**Table 4.** Inter-annual variability in maize yield (2005–11) under inoculation with *P. bilaiae*, in the large plot trials. Yield increase (%) is for inoculated crop relative to control (not inoculated)

Year	Yield (t/ha ± s.e.)	Range	Yield increase (% ±s.e.)	95% CI % increase*	Mean yield deviation (C^†^ *v*. I‡) (t/ha ± s.e.)	*Z*-test (C^†^ *v*. I‡)
2005	10·4 ± 0·33	2·5–14·4	3·5 ± 0·65	2·1–4·8	0·35 ± 0·071	<0·001
2006	10·3 ± 0·20	5·9–14·5	2·3 ± 0·47	1·4–3	0·22 ± 0·048	<0·001
2007	8·8 ± 0·29	5·1–14·1	5·2 ± 0·83	3·5–6·9	0·42 ± 0·066	<0·001
2008	9·8 ± 0·44	4·6–13·1	4·3 ± 0·63	3·0–5·6	0·38 ± 0·053	<0·001
2009	11·1 ± 0·32	5·7–15·4	3·4 ± 0·81	1·8–5·1	0·38 ± 0·083	<0·001
2010	10·1 ± 0·40	4·9–15·6	3·3 ± 0·83	1·6–4·9	0·32 ± 0·082	<0·001
2011	9·4 ± 0·38	5·8–14·3	4·2 ± 0·99	2·2–6·3	0·37 ± 0·087	<0·001

* Confidence interval.

† Control.

‡ Inoculated.

### Yield variance across sites

Maize yields measured in trials with large plots varied spatially. With inoculation, yield increased by 3–4% across the regions, regardless of overall yield levels in each state (Table 5). Field sites in North Dakota had the highest mean yield increase (4·2%) in response to *P. bilaiae* inoculation, and the lowest mean yield. Yield also increased due to inoculation for maize–maize and maize–soybean rotations in Minnesota. Rotation data were not available for all sites, but there were 24 sites where maize was the previous crop and 21 with soybean as the previous crop. The yield increase due to inoculation was 0·59 t/ha (5·6%) where maize followed maize and 0·31 t/ha (3·1%) where maize followed soybean. There was no significant difference in yield increase between the two crop rotations. To better understand the influence of inoculation at each yield level, all the small and large field trial data were partitioned into three yield groups; low (<6·3 t/ha), medium (6·3–12·6 t/ha) and high (>12·6 t/ha) (Table 6). Under this categorization, none of the small plots had yields under 6·3 t/ha, and there was no relationship between yield increase and mean yield attained under inoculation. In the large plots, however, the percent yield increase obtained with inoculation was highest in the lowest yield level under 6·3 t/ha, and decreased with increasing mean yield.

**Table 5.** Influence of inoculation with *P. bilaiae* on maize yield, by state in the large plot trials, 2005–2011, in units of t/ha± SE. 95% confidence intervals are provided

		With I*			Without I*			Deviation		
State	*n*	Mean (t/ha ± s.e.)	Lower CI†	Upper CI†	Mean (t/ha ± s.e.)	Lower CI†	Upper CI†	Mean (t/ha ± s.e.)	Lower CI†	Upper CI†
All	369	10·4 ± 0·13	10·1	10·6	10·1 ± 0·12	9·8	10·3	0·33 ± 0·026	0·03	0·51
Minnesota (MD)	101	11·1 ± 0·21	10·7	11·6	10·7 ± 0·21	10·3	11·1	0·44 ± 0·053	0·34	0·54
Nebraska (NE)	51	12·1 ± 0·29	11·4	12·6	11·7 ± 0·30	11·1	12·2	0·037 ± 0·080	0·2	0·53
South Dakota (SD)	72	10·1 ± 0·26	9·5	10·6	9·7 ± 0·26	9·2	10·2	0·39 ± 0·053	0·29	0·5
North Dakota (ND)	46	8·9 ± 0·22	8·5	9·3	8·5 ± 0·22	8·1	9	0·37 ± 0·074	0·2	0·5

* Inoculated.

† Confidence interval.

**Table 6.** Maize yield response to inoculation, relative to control plots, based on 2005–11 field trails comprising large (n = 369) and small (n = 92) sampling plots

Large plots			Small plots			
Yield level (t/ha)	*n*	% Increase (±s.e.)	Yield increase (t/ha ± s.e.)	*n*	% Increase (±s.e.)	Yield increase (t/ha ± s.e.)
Low <6·3	21	6·5 ± 1·2	0·34 ± 0·112		–	–
Medium (6·3–12·6)	264	3·4 ± 0·36	0·32 ± 0·031	66	2·2 ± 0·48	0·19 ± 0·048
High >12·6	54	2·6 ± 0·72	0·35 ± 0·070	26	0·8 ± 0·76	0·12 ± 0·077
*P*-value (Prob > F)		0·018	0·939		0·145	0·407

### Yield variance associated with soil pH and amount of applied phosphate

**Fig. 2.**
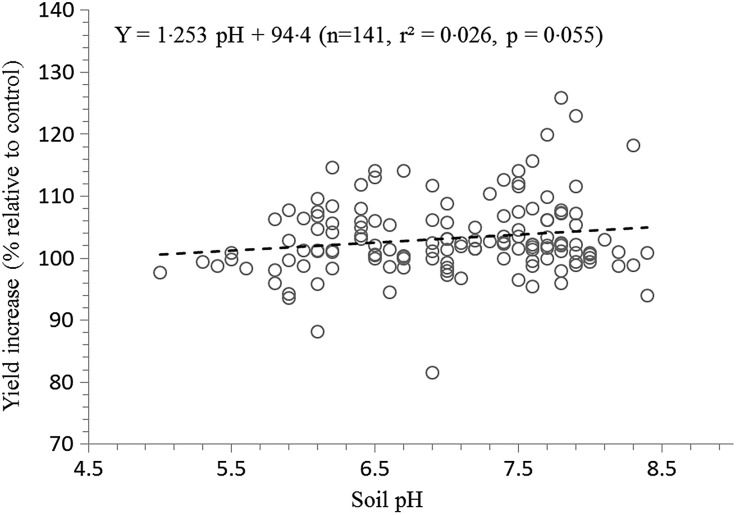
Effect of soil acidity on crop yield response to inoculation in terms of relative increase in maize yield compared to the control (*n* = 141).

**Fig. 3.**
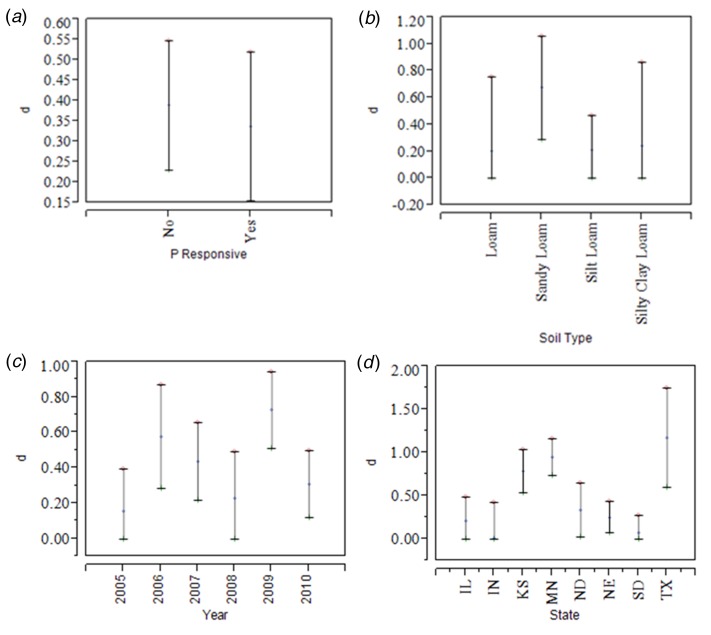
Standardized group mean difference (Cohen's d meta-analysis statistic) comparing the relative effect of year, location, phosphorus (P) response, and soil type on crop yield response to inoculation based on the field trial data. This provides a standardized approach to comparing these different effects. Mean estimates and confidence range of the effect size d (in standard deviation units) are provided and associated with: (a) P responsive and non-responsive sites, (b) variation in soil type, (c) sampling year, (d) sampling site location. Clay loam soil type and sites in Michigan were removed due to insufficient sample size. Values of *d* > 0·2, 0·5, 0·8 and 1·0 represent small, medium, large, and very large effects, respectively.

Maize yield increases with added P were observed in the field sites in 2006, in addition to interaction effects between both location and inoculant (*P* < 0·05), and location and phosphate fertilizer (*P* < 0·05) (Table 7). Inoculation treatments increased maize yield at both Minnesota sites (i.e., Jeffers and Fergus Falls), but not at the DeWitt site in Nebraska. There was a positive relationship between phosphate application and maize yield for DeWitt, but not at the other two sites. The DeWitt site also had the highest yields. There was no relationship between P applied and yield at the Minnesota sites. The effect of the amount of phosphate applied on the ability of the inoculant to increase maize yield) was examined in 2009 and 2010 (Table 8). No significant interaction effect was detected between phosphate applied and the influence of inoculation. Table 9 provides the fitted maize yield regression equations with added phosphate (P_2_O_5_) as the covariate for maize yield under inoculation relative to control for a selected set of fields. Best-fits were obtained for the DeWitt and Groom sites (highest *R*^2^, and smallest *P*-values). Across most of the sites, a robust linear or quadratic relationship between added phosphate and maize yield was not evident. To further examine the interaction between applied phosphate and influence of inoculation on maize yield, measured yield was grouped by soil P level (very low, low, medium and high) (Table 10). Reported estimates are based on soil test results for 116 of the large and 18 of the small-plot trials. The soil test data allowed for comparison of the efficacy of inoculation in soils with low, medium and high levels of soil P. No significant linear relationship was detected between maize yield and soil pH/acidity (in the range of 5·0–8·4), although results do indicate a trend for higher availability of soil P within the 6–8 pH range (Fig. 2). Computed values of the Cohen's effect size, meta-analysis statistic (d) that provides a standardized measure for comparing the observed variance on maize yield attributed to the known covariates of sampling year, site/location, soil type and soil P concentration, are summarized in Fig. 3.

**Table 7.** Effect of phosphate applied on maize inoculated with *P. bilaiae* in 2006. Numbers in brackets indicate sample size

	2006								
	DeWitt NE			Jeffers MN			Fergus falls MN		
P_2_O_5_ applied (kg/ha)	C (6) *	I (6) †	M (12)‡	C (6)*	I (6)†	M (12)‡	C (5)*	I (5)†	M (10)‡
0	14·2 ± 0·10	13·8 ± 0·18	14·0 ± 0·12	11·7 ± 0·19	12·0 ± 0·08	11·8 ± 0·11	8·3 ± 0·43	8·7 ± 0·25	8·4 ± 0·23
10	14·5 ± 0·09	14·2 ± 0·09	14·4 ± 0·08	11·3 ± 0·28	11·9 ± 0·23	11·6 ± 0·20	8·3 ± 0·24	8·8 ± 0·32	8·5 ± 0·20
20	14·1 ± 0·18	14·4 ± 0·30	14·2 ± 0·17	11·9 ± 0·16	12·1 ± 0·22	12·0 ± 0·13	8·6 ± 0·28	8·8 ± 0·26	8·7 ± 0·18
M	14·3 ± 0·08	14·2 ± 0·13		11·6 ± 0·13	12·0 ± 0·10		8·4 ± 0·18	8·7 ± 0·15	
	(18)	(18)		(18)	(18)		(15)	(15)	
*P*-values (Prob > F)									
P_2_O_5_ applied (kg/ha)	0·047			0·008			0·401		
*I*	0·414			0·022			0·022		
*P* × *I*	0·261			0·383			0·725		

* Control.

† Inoculated.

‡ Mean.

**Table 8.** Effect of phosphate applied on maize inoculated with *P. bilaiae* in 2009 and 2010

	2009										2010							
	Aurora NE		Centerville SD		Fergus falls MN		Garden City KS		Groom TX		Aurora NE		Bitton SD		University NE		Centerville SD	
P*	C†	I‡	C†	I‡	C†	I‡	C†	I‡	C†	I‡	C†	I‡	C†	I‡	C†	I‡	C†	I‡
	(6)	(12)	(6)	(1)	(6)	(12)	(6)	(12)	(6)	(12)	(6)	(12)	(6)	(12)	(6)	(12)	(6)	(12)
0	14·5 ± 0·29	14·6 ± 0·26	10·8 ± 0·54	11·6 ± 0·19	9·9 ± 0·22	11 ± 0·34	14·7 ± 0·33	15·1 ± 0·17	10·1 ± 0·32	13·1 ± 0·22	15·4 ± 0·27	15·5 ± 0·27	10·9 ± 0·16	11 ± 0·16	9·9 ± 0·27	10·2 ± 0·25	11·3 ± 0·17	11·5 ± 0·25
10	14·5 ± 0·17	14·6 ± 0·32	11·9 ± 0·21	12 ± 0·28	10·1 ± 0·38	11·4 ± 0·21	14·6 ± 0·28	15 ± 0·20	13·3 ± 0·33	13·5 ± 0·21	15·3 ± 0·17	16·1 ± 0·17	11·1 ± 0·23	11·2 ± 0·25	10 ± 0·41	10·1 ± 0·18	11·7 ± 0·24	11·4 ± 0·20
20	14·8 ± 0·23	14·9 ± 0·17	11·9 ± 0·35	12 ± 0·32	10·6 ± 0·41	11·1 ± 0·18	14·1 ± 0·53	14·7 ± 0·38	13·2 ± 0·22	13·2 ± 0·20	15·6 ± 0·18	15·4 ± 0·18	11 ± 0·40	11·2 ± 0·20	9·6 ± 0·19	9·9 ± 0·23	11·6 ± 0·19	12·1 ± 0·19
30	14·2 ± 0·19	14·6 ± 0·16	12·2 ± 0·47	12·3 ± 0·32	11·1 ± 0·64	11·7 ± 0·30	14·3 ± 0·33	15 ± 0·32	13 ± 0·24	13 ± 0·21	15·4 ± 0·19	15·4 ± 0·19	11·3 ± 0·30	11·5 ± 0·25	9·5 ± 0·16	9·2 ± 0·26	11·5 ± 0·68	11·7 ± 0·24
M§	14·5 ± 0·23	14·7 ± 0·21	11·7 ± 0·21	12 ± 0·14	10·4 ± 0·15	11·3 ± 0·11	14·4 ± 0·26	15 ± 0·25	12·4 ± 0·14	13·2 ± 0·10	15·4 ± 0·14	15·6 ± 0·08	11·1 ± 0·22	11·2 ± 0·21	9·7 ± 0·19	9·8 ± 0·15	11·5 ± 0·17	11·7 ± 0·12
*P*-values (Prob > *F*)																		
P*	0·048		0·066		0·370		0·637		<0·001		0·460		0·387		0·859		0·619	
I‡	0·200		0·325		<0·001		0·003		<0·001		0·379		0·300		0·344		0·451	
P*XI‡	0·785		0·701		0·304		0·879		<0·001		0·324		0·960		0·710		0·731	

* Phosphorus.

† Control.

‡ Inoculant.

§ Mean.

**Table 9.** Site-specific fitted regression equations for maize yield (t/ha) with added phosphate (P_2_O_5_) as a covariate. (−) indicates that no polynomial fit to the data was obtained

Location	Year	Fitted equation for yield	Fit type	*P* value	*R*^2^
DeWitt, NE	2006	*Y* = 14·6–0·003 × P_2_O_5_–0·004 (P_2_O_5_–10)^2^	Quadratic	**0·030**	**0**·**279**
Jeffers, MD	2006	*Y* = 11·5 + 0·014 × P_2_O_5_	–	0·416	0·042
Fergus Falls, MD	2006	*Y* = 8·2 + 0·016 × P_2_O_5_	–	0·483	0·038
Aurora, NE	2009	*Y* = 14·6–0·008 × P_2_O_5_	–	0·401	0·032
Centerville, SD	2009	*Y* = 11·1+ 0·042 × P_2_O_5_	Linear	**0·033**	**0·191**
Fergus Falls, MN	2009	*Y* = 9·8 + 0·040 × P_2_O_5_	Linear	0·043	0·173
Garden City, KS	2009	*Y* = 14·6–0·015 × P_2_O_5_	–	0·365	0·037
Groom, TX	2009	*Y* = 11·1 + 0·084 × P_2_O_5_	–	**0**·**001**	**0**·**419**
Aurora, NE	2010	*Y* = 15·45 + 0·002 × P_2_O_5_	Linear	0·401	0·001
Britton, SD	2010	*Y* = 10·9 + 0·010 × P_2_O_5_	–	0·431	0·028
Centerville, SD	2010	*Y* = 11·4 + 0·006 × P_2_O_5_	–	0·704	0·007
Nebraska, NE	2010	*Y* = 9·9–0·014 × P_2_O_5_	–	0·300	0·053

**Table 10.** Maize yield increases in large versus small plots, by soil phosphorus concentration level under inoculation with *P. bilaiae*, 2005–10

	Large plots		Small plots	
Phosphorus level	*n*	Yield increase (t/ha ± s.e.)	*n*	Yield increase (t/ha±s.e.)
Very low	35	0·33 ± 0·09	5	0·32 ± 0·110
Low	48	0·31 ± 0·08	7	0·21 ± 0·093
Medium	18	0·30 ± 0·13	4	−0·11 ± 0·123
High	16	0·00 ± 0·14	2	n/a
*P*-value (Prob > *F*)	0·187		0·004	

## DISCUSSION

Previously, *P. bilaiae* has reportedly increased P uptake, and subsequently increased growth and yield of various crop species including wheat, peas and canola, under both greenhouse and field conditions in a range of different soils (Chambers & Yeomans 1990, 1991; Gleddie *et al.* 1991; Gleddie 1993, Leggett *et al.* 1993; Beckie *et al.* 1998). Evidence that *P. bilaiae* inoculation also increases maize yields with greater response to inoculation occurring in the larger demonstration trials is provided in the current work. Beckie *et al.* (1998) also noted differences between small and large plot yield increases following *P. bilaiae* inoculation of alfalfa, where the increases in yield and P fertilizer replacement (plant P content) value attributed to *P. bilaiae* inoculation were greater in large plots. In the present study, the inoculant was most effective at increasing yield at sites that had low soil phosphorus levels for both small and large plots. At higher levels of soil phosphorus, yield increase attributed to inoculation was greater in the large plots than in the small plots. Interestingly, yield increase differences between small and large plots were not observed in winter wheat evaluation trials conducted by Yan *et al.* (2002). Greater yield response in the large plot trials in the present study could be partially explained by a negative edge effect due to a higher density of *P. bilaiae* in the centre of each plot, leading to greater phosphate solubilization and uptake in that region compared to the plot edges. Findings of Beckie *et al.* (1998) suggest also that observed differences in response to inoculation between small and large plots might be related to management practices, such as seeding into standing stubble, precision fertilizer placement, or growing a companion crop. Mallarino (1996) assessed patterns of spatial P variability at eight zero-tilled maize (*Zea mays* L.) fields, measuring high random and spatially structured variability across the maize fields. The spatially structured variability was best explained as arising from repeated banded-fertilizer applications of P, at small spatial scales, and broadcast fertilization with commercial bulk spreaders at larger spatial environments. Soil type and field topography probably also had an influence on soil phosphorus availability, mediating yield increases under inoculation between the large and small plot trials.

Trials with other crops have shown a distinct relationship between soil P and the effect of inoculation with *P. bilaiae*, indicating greater benefits from inoculation in environments with limited P availability. *P. bilaiae* solubilizes inorganic phosphorus and can release large amounts of P from rock phosphate (Asea *et al.* 1988). In wheat, inoculation with *P. bilaiae* increased yield at the lowest rates of applied phosphate, and the positive effect of inoculation decreased when P was no longer a limiting factor on crop growth (Gleddie *et al.* 1991). In canola, application of phosphate or inoculation with *P. bilaiae* increased grain yield at P-responsive but not at non-responsive sites (Gleddie 1993). *Penicillium bilaiae* increased tissue P concentration in wheat and flax in the absence of added phosphate (Chambers & Yeomans 1991). Canola inoculated with *P. bilaiae* in a greenhouse trial had higher tissue P contents than un-inoculated plants in treatments with either no fertilization or where rock-P or soluble phosphate was supplied at low rates, but not at higher rates where P fertilizer alone was able to provide enough P to plants for maximal growth (Kucey 1983, 1987; Kucey & Leggett 1989). In addition, ^32^P dilution showed that inoculation with *P. bilaiae* increased P concentration in plants and that a higher proportion of the plant P was derived from sources unavailable to un-inoculated control plants (Asea *et al.* 1988; Chambers & Yeomans 1991).

In the field studies analysed in the current paper, *P. bilaiae* inoculant increased maize yield in both P responsive and non-responsive soils, regardless of the amount of applied phosphate fertilizer. Maize has a high demand for P and meeting this need is directly related to the soil solution P source, which must be adequately replenished throughout the growing season. For example, if biomass production was limited by drought, plant P accumulation may be *sink*-limited, but if the concentration of the soil solution is not replenished rapidly enough, plant P accumulation may be source-limited. If the available P is adequate for the maize plant, adding more P to the source (soil solution) does not impact the plant's growth. The phosphate inoculant acts differently from rhizobial inoculants, which fix nitrogen from sources other than what is contained in the soil or applied as fertilizer. The inoculant used in the present study does not provide P; it only makes P in soil more available. The *P. bilaiae* inoculant solubilizes plant-unavailable soil phosphate, increasing the P available for plant uptake (Asea *et al.* 1988; Chambers & Yeomans 1991), which can lead to increased plant uptake and an increase in plant P content. Adequate P in the soil (available sources) and fertilizer applications have to equal or exceed the crop's seasonal need for optimal growth and yield. Sub-optimal soil P levels could explain why yield increases obtained with *P. bilaiae* inoculation varied so much among years and trial sites. To fully understand the underlying factors for the variable performance of inoculation in different trials one would need to know the amount of P taken up by the crop in both inoculated and control plots. Unfortunately, P concentration in plant tissue was not measured in the trials analysed in the present study.

Some of the yield gain observed in the field trials may also be due to other growth-promoting effects of *P. bilaiae* beyond phosphate solubilization. For example, rhizosphere micro-organisms can increase root growth or stimulate root hair development through the production of phytohormones, or alteration of the root surface membrane potential and subsequent effects on the efflux of H^+^ ions (Richardson & Simpson 2011). *Penicillium bilaiae* can exert direct effects on plant roots by stimulating root hair development, which could increase plant growth by meeting the plants P requirements more consistently through increased absorptive capacity of roots (Gulden & Vessey 2000; Vessey & Heisinger 2001). Root hairs are involved in P uptake by extension of the P depletion zone and the increased ability of the plant to replenish the solution P, which is especially important for a crop such as maize. The impact of root hairs is well documented and may contribute up to 90% of the total uptake by the root system in low-P soil (Föhse *et al.* 1991; Gahoonia & Nielsen^1998^). Such effects could explain the increase of maize yield when using the *P. bilaiae* inoculant in soils with no obvious P limitation.

Very few studies published to date include such a large multi-year and multi-site representative set of field-scale trial data on crop yield and its variability, partly because in environmental studies no single fixed experimental design can be set and the high spatial variability of soil parameters makes it challenging to meet the requirements of traditional statistical methods designed to compare treatment means (Gili *et al.* 2013). The Cohen's effect-size results computed in the analysis of the current data indicate that maize yield variance due to inoculation with *P. bilaiae* is relatively uniform across sampling years. Results also show, however, that yield variance is more variable among the sites in Texas and Illinois, which show the largest confidence interval (CI) range, and the lowest and highest effect size, respectively. High variability could be explained by low numbers of available soil P test results and a high number of non-replicated studies, especially in loam and silty loam soils. In general, effect size (d) decreased in the following order: site location, soil type, sampling year/inter-annual variability and soil P concentration. It has been suggested that *P. bilaiae* has a more consistent positive measurable impact on yield in neutral to alkaline soils (Leggett *et al.* 2001). In maize, however, the relationship between efficacy and soil pH was weak, suggesting that environmental factors other than soil acidity mediate efficacy. Richardson (2001) emphasized that poor understanding of the interactions between physical and chemical characteristics of soil and P mobilization is a major limitation to the application of phosphorus solubilizing inoculants. While the current findings are limited with regard to the measurable influence of inoculation on maize yield in relation to a broad set of other possible soil and environmental covariates/conditions, the results reported in the current paper nonetheless help to guide and rank the covariates exhibiting the largest variation and therefore the largest potential gains in statistical accuracy on maize yield response to inoculation. Specifically, stratified sampling design by site location and transect-based variation in soil characteristics is better able to capture multi-scale variation. Also, more data on soil P and plant P uptake could increase accuracy and reliability on yield response effects. A spatially stratified trial design could involve additional soil, plant and climate measurements, such as soil water stress index, growing degree day and maize heat units that account for variability and mediate factors controlling inoculation efficiency and associated yield response.
